# Upregulation of programmed death ligand-1 in tumor-associated macrophages affects chemotherapeutic response in ovarian cancer cells

**DOI:** 10.1371/journal.pone.0277285

**Published:** 2023-02-09

**Authors:** Yong Soo Jang, Tae Wan Kim, Jae Sung Ryu, Hye Jeong Kong, Si Hyeong Jang, Gye Hyun Nam, Jae Hoon Kim, Seob Jeon

**Affiliations:** 1 Department of Obstetrics and Gynecology, College of Medicine, Soonchunhyang University Cheonan Hospital, Cheonan, Korea; 2 Soonchunhyang Innovative Convergence Research Center, Soonchunhyang University Cheonan Hospital, Cheonan, Korea; 3 Department of Medical Life Science, Soonchunhyang University, Asan, Korea; 4 Department of Pathology, College of Medicine, Soonchunhyang University Cheonan Hospital, Cheonan, Korea; 5 Department of Obstetrics and Gynecology, College of Medicine, Soonchunhyang University Bucheon Hospital, Bucheon, Korea; 6 Department of Obstetrics and Gynecology, Gangnam Severance Hospital, Yonsei University College of Medicine, Seoul, Korea; Babasaheb Bhimrao Ambedkar University, INDIA

## Abstract

To better understand the mechanism of chemoresistance in ovarian cancer cells, we aimed to investigate the influence of macrophages on the tumor cell response to carboplatin and identify the genes associated with chemoresistance. We mimicked the tumor microenvironment (TME) using a co-culture technique and compared the proliferation of ovarian cells with and without macrophages. We also examined M1 and M2 marker expression and the expression of key TME genes. Post the co-culture, we treated ovarian cancer cells with carboplatin and elucidated the function of programmed death–ligand 1 (PD-L1) in carboplatin chemoresistance. We investigated CD68 and PD-L1 expression in normal and cancerous ovarian tissues using immunohistochemistry (IHC). Finally, we analyzed the association between CD68 or PD-L1 expression and survival outcomes. Inducible nitric oxide synthase (*iNOS*) was downregulated, while the gene expression of M2 macrophage markers was increased in ovarian cancer cells. *PD-L1*, vascular endothelial growth factor (*VEGF*), Interleukin (*IL)-6*, *IL-10*, *IL-12*, signal transducer and activator of transcription 3 (*STAT3*), B-cell lymphoma 2 (*BCL2*), multidrug resistance 1 (*MDR1*), and colony stimulating factor 1 (*CSF-1*) were upregulated. Notably, PD-L1 was upregulated in both the ovarian cancer cells and macrophages. Ovarian cancer cells co-cultured with macrophages exhibited statistically significant carboplatin resistance compared to single-cultured ovarian cancer cells. PD-L1 silencing induced chemosensitivity in both types of co-cultured ovarian cancer cells. However, IHC results revealed no correlation between PD-L1 expression and patient survival or cancer stage. CD68 expression was significantly increased in cancer cells compared to normal or benign ovarian tumor cells, but it was not associated with the survival outcomes of ovarian cancer patients. Our study demonstrated that ovarian cancer cells interact with macrophages to induce the M2 phenotype. We also established that PD-L1 upregulation in both ovarian cancer cells and macrophages is a key factor for carboplatin chemoresistance.

## Introduction

Epithelial ovarian cancer is the leading cause of cancer-related mortality in women with gynecologic cancers. In 2020, an estimated 313,959 new cases of ovarian cancer and 207,252 ovarian cancer-related deaths were reported worldwide [1]. Current standard treatments for ovarian cancer are based on cytoreductive surgery followed by platinum-based chemotherapy, such as carboplatin. Although many patients respond to initial chemotherapy, intrinsic or acquired chemoresistance remains a major challenge.

Tumor-associated macrophages (TAMs) are a heterogeneous population of myeloid cells in the tumor microenvironment (TME). Compelling evidence has suggested that TAMs promote key processes associated with tumor progression, metastasis, angiogenesis, and immunosuppression in various malignancies, including breast, ovarian, hepatocellular, and gastric cancers [2–5]. There are two distinct macrophage polarization states based on their activation—classically activated macrophages (M1 macrophages) and alternatively activated macrophages (M2 macrophages) [6]. TAMs in the TME have a predominantly M2-like phenotype in response to interleukin (IL)-6 and macrophage colony-stimulating factor (M-CSF) [7, 8]. M2 macrophages in ovarian tumors have been confirmed by several studies, demonstrating a positive correlation between their high levels in tumors and the low overall survival of patients [9–11]. Chemotherapeutic agents, such as cisplatin and carboplatin, increase the potency of tumor cell lines to induce the M2 macrophage phenotype; this is possibly an indirect mechanism of chemoresistance [12].

Programmed death-1 (PD-1) is an immune checkpoint receptor that is upregulated in activated T-cells for immune tolerance. However, tumor cells overexpress the PD-1 substrate programmed death–ligand 1 (PD-L1) to suppress the immune system. It is expressed in both cancer cells and macrophages. The upregulation of PD-L1 expression in non-small cell lung cancers (NSLC) promotes a chemoresistance response [13]. Although PD-L1 expression in tumor cells is related to chemoresistance, little is known about the role of PD-L1 in the interaction between tumor cells and macrophages in the TME. An understanding of the regulatory mechanisms involved in PD-L1 expression, especially TAM-induced regulation in the TME, will be helpful in clinical treatment.

In this study, we investigated PD-L1 expression and the interaction between macrophages and tumor cells using the Transwell co-culture assay. We examined the role of M2 macrophage polarization and PD-L1 expression in carboplatin chemoresistance associated with ovarian cancer cells. We also investigated the relationship between TAM infiltration or PD-L1 expression and patient survival using immunohistochemical (IHC) analysis.

## Materials and methods

### Cell lines and culture

We obtained the human ovarian cancer cell line SKOV3, human monocyte cell line THP-1, mouse ovarian cancer cell line ID8, and mouse macrophage cell line RAW264.7 from the Korean Cell Line Bank (Seoul, Korea). We cultured these cells in an RPMI 1640 medium (WELGENE, Gyeongsan, Korea), containing 10% fetal bovine serum (FBS; YounginFrontier, Seoul, Korea) and 1% antibiotic solution (ABS; Corning Inc, NY, CA, USA), at 37°C in a humid atmosphere of 5% CO_2_. Moreover, we induced the THP-1 cells to differentiate into macrophages by adding 320 nM of 12-O-tetradecanoylphorbol-13-acetate (PMA) to the culture medium. Green fluorescence protein (GFP) was transfected into the SKOV3 cell line using a pCDH vector, and the transfected cells were selected with 3 μg/mL of puromycin.

### Patients and tumor specimens

We obtained tumor samples from 47 patients with ovarian cancer and 35 patients with benign ovarian tumor, confirmed pathologically, who underwent surgery at Soonchunhyang University Cheonan Hospital. We used an additional seven normal ovarian tissue samples from our institution and 38 tumor samples from the Human Material Bank in Gangnam Severance Hospital. Ethical approval for this study was obtained from the Institutional Review Board (SCHCA2016-02-023). Informed written consent was acquired from all the patients.

### Immunohistochemical staining of PD-L1 and CD68

We assessed PD-L1 expression via immunohistochemical staining. We sectioned 4 μm thick paraffin-embedded blocks of patient tissues, dried the slides for one day, and kept them at 60°C for an hour. Antigen retrieval was performed using 3% H_2_O_2_ and a 95°C antigen retrieval buffer. The tissue sections were permeabilized with a 0.2% Triton solution and blocked with 5% bovine albumin serum in PBS for 15 min. They were then incubated overnight with a rabbit polyclonal primary PD-L1 antibody (1:100, Thermo Fisher, PA5-20343) at 4°C. Thereafter, the sections were incubated with goat anti-rabbit IgG (H+L) and horseradish peroxidase-tagged secondary antibodies (1:100, Thermo Fisher, 31460) for 1 h at 23°C. The sections were then stained with DAB (3,3’-diaminobenzidine, VECTORLABS, SK-4100) and counterstained with 50% hematoxylin for 30 s. The slides were subsequently dried at 37°C for 1 h and mounted with the Eukitt® Quick-hardening mounting medium (Sigma-Aldrich, 03989-100ML). The slides were interpreted twice by two trained pathologists under an optical light microscope (Leica microsystems DMi1, Wetzlar, Germany). The CD68^+^ sections were counted in normal, benign, and ovarian cancer tissues and classified into low and high subsets based on the median value of the CD68^+^ counts in ovarian cancer sections. The PD-L1 sections were quantified based on the percentage of stained cells as follows: 0 staining intensity, no stained cancer cells; +1, <33% positively stained cancer cells; +2, 33–66% positively stained cancer cells; and +3, >66% positively stained cancer cells.

### Transwell co-culture assay

We used ID8, RAW264.7, SKOV3, and THP-1 cells for the Transwell co-culture assay. We seeded 1×10^4^ ID8 (or SKOV3) cells/well into a 24-well culture plate and 1×10^4^ RAW264.7 (or THP-1) cells onto polycarbonate Transwell inserts in 24-well plates (pore size 8.0 μm; Corning, 3422). These cells were grown in an RPMI 1640 medium containing 10% FBS and 1% ABS at 37°C in a humid atmosphere of 5% CO_2_.

### Wound healing assay

The wound healing capacity of the cells was measured using culture inserts (Ibidi, 81176). We cultured 1×10^5^/200 μL of ID8 cells in culture inserts for 24 h. Subsequently, we created a cell-free gap of 500 μm by removing the culture inserts. The old medium was removed, and cells were either treated with a co-culture (to recreate the TME) or a single-culture soup. The optical images were visualized after 0, 8, 16, and 24 h of wound creation. The percentage of wound closure fields was calculated using the Image J software (NIH, 1.52a).

### Reverse transcription-polymerase chain reaction (RT-PCR)

We seeded and incubated 1×10^4^ ID8 (or SKOV3) cells/well into 24-well plates and 1×10^4^ RAW264.7 (or THP-1) cells onto polycarbonate Transwell inserts in 24-well plates for 72 h. Subsequently, we removed the supernatants and transferred the inserts to a new 24-well plate. We added 1 mL RiboEx (Geneall, 305–101, Seoul, Korea) to each well, according to the manufacturer’s instructions, and incubated the plate for 5 min at room temperature. Next, we added 200 μL chloroform per 1 mL of RiboEx. This mixture was shaken vigorously for 15 s, incubated for 2 min at room temperature, and centrifuged at 12,000 × *g* at 4°C for 15 min. We transferred the aqueous phase to a fresh tube and added 1 volume of RB1 Buffer (Geneall) to it; thereafter, we mixed the contents thoroughly by inverting the tube. Subsequently, we transferred up to 700 μL of the mixture to a mini-column type F and centrifuged it at ≥10,000 × *g* for 30 s at room temperature. We added 500 μL of SW1 Buffer (Geneall) to the mini-column and centrifuged it at ≥10,000 × g for 30 s at room temperature. Next, we added 500 μL RNW Buffer (Geneall) to the mini-column and centrifuged it at ≥10,000 × *g* for 30 s at room temperature. We then centrifuged it at ≥10,000 × *g* for an additional 1 min at room temperature to remove the residual wash buffer. We transferred the mini-column to a new 1.5 mL microcentrifuge tube and added 50 μL nuclease-free water to the center of the mini-column membrane. The column was kept stationary for 1 min and then centrifuged at ≥10,000 × *g* for 1 min at room temperature. The extracted RNA was immediately reverse-transcribed to cDNA using the ReverTra Ace® qPCR RT Master Mix kit (TOYOBO, FSQ-201). Quantitative RT-PCR was performed using the SYBR Green Real-time PCR Master Mix kit (TOYOBO, QPK-201); the primer pairs are listed in Table 1. The PCR cycle included one cycle at 95°C for 1 min, followed by 32 cycles at 95°C for 15 s, 60°C for 15 s, and 72°C for 25 s. We used the Quantitative RT-PCR protocol from the CFX96™ System (BIO-RAD, 3600037). The PCR cycle included one cycle denaturation at 95°C for 1 min, followed by 32 cycles at denaturation 95°C for 15 s, primer annealing 60°C for 15 s, and elongation 72°C for 25 s.

**Table 1:** RT-PCR primer pairs designed based on sequence information obtained from the Ensembl genomic database (ensembl.org).

**Real time polymerase chain reaction Primer list**				
**Species**	**Gene name**	**Forward (5’-3’)**	**Reverse (5’-3’)**	**Ensembl id**
Human	*STAT3*	GAG CAG AGA TGT GGG AAT GG	TCT TGG GAT TGT TGG TCA GC	ENSG00000168610
	*VEGF*	ACT TTC TGC TGT CTT GGG TG	ATG TAC TCG ATC TCA TCA GGG	ENSG00000112715
	*CSF-1*	TGC GCT TCA GAG ATA TCT CC	GCT GTT GTT GCA GTT CTT GC	ENSG00000184371
	*PD-L1*	GTT GTG GAT CCA GTC ACC TC	TGT TGT GTT GAT TCT CAG TGT G	ENSG00000120217
	*CD206*	AGT CAG ATC ACA CAG CAT GG	ATT TAT CCA CAG CCA CGT CC	ENSG00000260314
	*IL-12A*	TGG CCT CCA GAA AGA CCT C	GCA TGA AGA AGT ATG CAG AGC	ENSG00000168811
	*IL-12B*	ATT GAG GTC ATG GTG GAT GC	TTT CTC TCT TGC TCT TGC CC	ENSG00000113302
	*Arginase1*	GGA CCC ATC TTT CAC ACC AG	CAA GTC CGA AAC AAG CCA AG	ENSG00000118520
	*iNOS*	TGC TGT GCT CCA TAG TTT CC	TCT CTT CTC TTG GGT CTC CG	ENSG00000007171
	*IL-6*	CAA TGA GGA GAC TTG CCT GG	GCG CAG AAT GAG ATG AGT TG	ENSG00000136244
	*IL-10*	CCA AGC TGA GAA CCA AGA CC	AGA TGT CAA ACT CAC TCA TGG C	ENSG00000136634
	MDR1	GGT TCT GGG AAG ATC GCT AC	ACC GGA AAC ATC CAG CAT AG	ENSG00000085563
	BCL2	GGA GGA TTG TGG CCT TCT TTG	CCC AGC CTC CGT TAT CCT G	ENSG00000171791
	ALDH1	GTT CCT GGT TAT GGG CCT AC	ATG CGG CTA TAC AAC ACT GG	ENSG00000165092
	GAPDH	TGT TCG TCA TGG GTG TGA AC	GCA GGG ATG ATG TTC TGG AG	ENSG00000111640
**Species**	**Gene name**	**Forward (5’-3’)**	**Reverse (5’-3’)**	**Ensembl version**
Mouse	*STAT3*	ACC GTG AGC CTA CAA CCA TC	GAT CGC TTA CTC TCC GCA TC	ENSG00000168610
	*VEGF*	AGG ATG TCC TCA CTC GGA TG	TTG GAA CCG GCA TCT TTA TC	ENSG00000112715
	*CSF-1*	GCA GTA CCA CCA TCC ACT TGT A	GTG AGA CAC TGT CCT TCA GTG C	ENSG00000184371
	*PD-L1*	TGC TGC ATA ATC AGC TAC GG	GCT GGT CAC ATT GAG AAG CA	ENSG00000120217
	*CD206*	GGC AGG ATC TTG GCA ACC TAG TA	CCT TTC TTC CGA CTC TTC ACC C	ENSG00000260314
	IL-12	GAG GAG GGG TGT AAC CAG AAA GG	GCA TCC TAG GAT CGG ACC CTG	ENSG00000168811
	Arginase1	ACC TGG CCT TTG TTG ATG TCC CTA	AGA GAT GCT TCC AAC TGC CAG ACT	ENSG00000118520
	iNOS	CTC ACT GGG ACA GCA CAG AA	TGG GTC CTC TGG TCA AAC TC	ENSG00000007171
	IL-6	TG CCA CCT TTA CTG ATG GGA G	CA AGC CAG GGG AGA TGC TTT	ENSG00000136244
	IL-10	TCT CCA GGG CAG CCT AAG TA	CTG CAG GTG TAC CCC AAG TT	ENSG00000136634
	MDR1	AGC TGG TTC GAT GAC CAT AAG	CTC AAG CTG TTT CTT GTC CTT C	ENSG00000085563
	BCL2	CTT CGC AGA GAT GTC CAG TC	AGA TGC CGG TTC AGG TAC TC	ENSG00000171791
	ALDH1	ACT TGG CCT TCA GAG AAC GA	TAG ACC CCA GGC TAC CAT TG	ENSG00000165092
	GAPDH	TCA AGG CCG AGA ATG GGA AG	CTA AGC AGT TGG TGG TGC AG	ENSG00000111640

### Small interfering RNA (siRNA) transfection

We seeded 2×10^5^ SKOV3 cells per 60 mm^2^ culture dish. Once the cells reached 50–60% confluency, the medium was changed to a serum-free RPMI 1640 medium, and the cells were cultured for 24 h. Subsequently, 25 μL of 3 μg/μL human *PD-L1* small interfering RNA (siRNA; Sigma, Burlington, USA) was mixed with 75 μL X-tremeGENE™ siRNA Transfection Reagent (Sigma) and added to the cells.

We incubated 1×10^6^ THP-1 cells per T75 culture flask for 24 h. Subsequently, the medium was changed to a serum-free RPMI 1640 medium containing 320 μM PMA (Merck, Germany), and the cells were cultured for 72 h to induce polarization. Thereafter, the medium was changed to an RPMI 1640 medium containing 10% FBS and 1% ABS, and the cells were cultured for 24 h. This medium was then changed to a serum-free RPMI 1640 medium, and the cells were cultured for a further 24 h. Thereafter, 30 μL of 3 μg/μL human PD-L1 siRNA (Sigma) was mixed with 90 μL transfection reagent and added to the cells.

### Immunofluorescence staining and confocal microscopy

We reproduced the co-cultivation conditions using soup change. The co-culture soup was obtained from co-cultured T75 flasks (SPL Ltd., Pocheon, Korea) that were seeded with 5×10^5^ SKOV3-GFP cells and 5×10^5^ PMA-treated THP-1 cells. The single-culture soup was obtained from a single-cultured T75 flask that was seeded with only SKOV3-GFP cells. After three days, we collected the co-culture and single-culture soups from each T75 flask. Thereafter, we cultured 1×10^5^ SKOV3-GFP and THP-1 cells/well in four-well chambered culture slides (SPL Ltd.) for 24 h. The old media was removed, and the cells were treated with co-culture and single-culture soups for 48 h. Thereafter, all the media was removed, and the cells were washed thrice with PBS and fixed using 4% formaldehyde for 15 min at room temperature. Following this, all the supernatants were removed, and the cells were blocked for 60 min with a solution containing 94.9% PBS, 4.8% FBS, and 0.3% Triton-X 100. The cells were incubated overnight with a rabbit polyclonal primary PD-L1 antibody (1:100, Thermo Fisher, PA5-20343) at 4°C. Subsequently, the cells were incubated with Alexa Fluor® Plus 594-tagged donkey anti-rabbit IgG (H+L) highly cross-adsorbed polyclonal secondary antibody (1:1000, Thermo Fisher, A32754) for 1 h at room temperature. We then performed Hoechst staining and compared the relative fluorescence intensities obtained under different culture conditions under a confocal laser scanning microscope (ZEISS LSM 710, ZEISS, Berlin, Germany).

### Cell viability test after carboplatin treatment

We seeded 1×10^4^ ID8 (or SKOV3) cells/well in 24-well plates and RAW264.7 (or THP-1) cells onto polycarbonate Transwell inserts in 24-well plates. We evaluated the effect of co-culturing RAW264.7 (or THP-1) with ID8 (SKOV3) cells upon carboplatin treatment by adding varying carboplatin concentrations (0, 50, 100, and 200 μM) to each well and incubating for 48 h. Thereafter, we evaluated the cell viability by performing the EZ-Cytox cell viability assay. For this, we incubated the cells with a non-serum medium (750 μL) containing 10% EZ-Cytox reagent for 4 h at 37°C. The absorbance intensities of the wells were measured at 579–630 nm using a plate reader (Spectrophotometer 1510, Thermo Fisher Scientific, Vantaa, Finland). The percent viability of the cells was calculated as the percent color formation in the experimental wells relative to the control wells. This experiment was performed in triplicate.

### Western blotting

Cells were washed in phosphate-buffered saline (PBS) and lysed in Pro-Prep™ protein extraction solution (iNtRON, Seongnam, Korea). The protein concentration was determined by the bicinchoninic acid (BCA) assay using an xMark Microplate Absorbance 208 Spectrophotometer (Bio-Rad Laboratories, Inc., Hercules, CA, USA). The lysate was centrifuged and the protein in the supernatant was denatured by boiling for 10 min at 100°C. Equal quantities of protein (30 μg/lane) were resolved by 10% sodium dodecyl sulfate–polyacrylamide gel electrophoresis (SDS-PAGE) and transferred onto an Immobilon polyvinylidene difluoride membrane (Millipore, Billerica, MA, USA). The membrane was then blocked for 1 h in 5% skim milk. One membrane was incubated overnight at 4°C with anti-human B-actin polyclonal antibody (Santa Cruz Biotechnology, sc-47778) diluted 1:500 and incubated with the secondary antibody for 2 h at room temperature. The other membranes were incubated overnight at 4°C with anti-human PD-L1 polyclonal antibody (Thermo Fisher, PA5-20343) diluted 1:500 and incubated with the secondary antibody for 2 h at room temperature. The signal was detected using ECL western detection reagents (Advansta, Menlo Park, CA, USA) and a Molecular Imager Chemi-luminescence Bioimaging Instrument (Cellgenetek, Deajeon, Korea).

### Statistical analysis

The wound closure percentage was statistically analyzed using the Student’s *t*-test and the Statistical Package for the Social Sciences v19.0 (SPSS; IBM, Chicago, IL, USA). However, the rest of the data were analyzed using SPSS v23.0. The *t*-test was used to compare the expression of CD68 between normal or benign and cancer tissues. Survival analyses were performed using the Kaplan-Meier method, and differences between groups were tested using the log-rank test. Progression-free survival (PFS) was defined as the time interval between the surgery and tumor recurrence or the last follow-up. Overall survival (OS) was defined as the time interval between the diagnosis of the disease and death or the last follow-up. These independent prognostic factors, i.e., PFS and OS, were examined via multivariate analysis using the Cox regression model. Differences were considered statistically significant at p-values < 0.05.

## Results

### Baseline clinical characteristics

The baseline clinicopathological characteristics of all 85 patients with ovarian cancer are summarized in Table 2. The median age at diagnosis was 52.6 years (17–76 age range). Approximately half of the patients had serous carcinoma (n = 48, 56%), followed by clear cell carcinoma (n = 15, 18%), mucinous carcinoma (n = 12, 14%), and endometrioid carcinoma (n = 10, 12%). At the time of diagnosis, most patients had FIGO stage III (n = 37, 43%), followed by stages I (n = 32, 38%), II (n = 9, 11%), and IV (n = 7, 8%). Three-fourths of the cases (n = 67, 79%) had PD-L1 low tumors (i.e., the intensity of PD-L1 expression was less than 2+). Recurrence was observed in 35 patients (41%), and the median value for progression-free survival was 20.48. In all patients, the median overall survival was 51.49.

**Table 2:** Characteristics of patients.

Patients (*n* = 85)		
**Age (median, range)**	52.6, 17–76 years	
**Histopathology**	Serous	48 (56%)
	Mucinous	12 (14%)
	Endometrioid	10 (12%)
	Clear cell	15 (18%)
**FIGO Stage**	Stage I	32 (38%)
	Stage II	9 (11%)
	Stage III	37 (43%)
	Stage IV	7 (8%)
**Intensity of PD-L1 expression**	Low (0,1+)	67 (79%)
	High (2+,3+)	18 (21%)
**Recurrence of disease**	35 (41%)	
**Progression-free survival (median, range)**	20.48 (2–80)	
**Overall survival (median, range)**	51.49 (8–107)	

### Influence of macrophages on tumor cells

**Fig 1:**
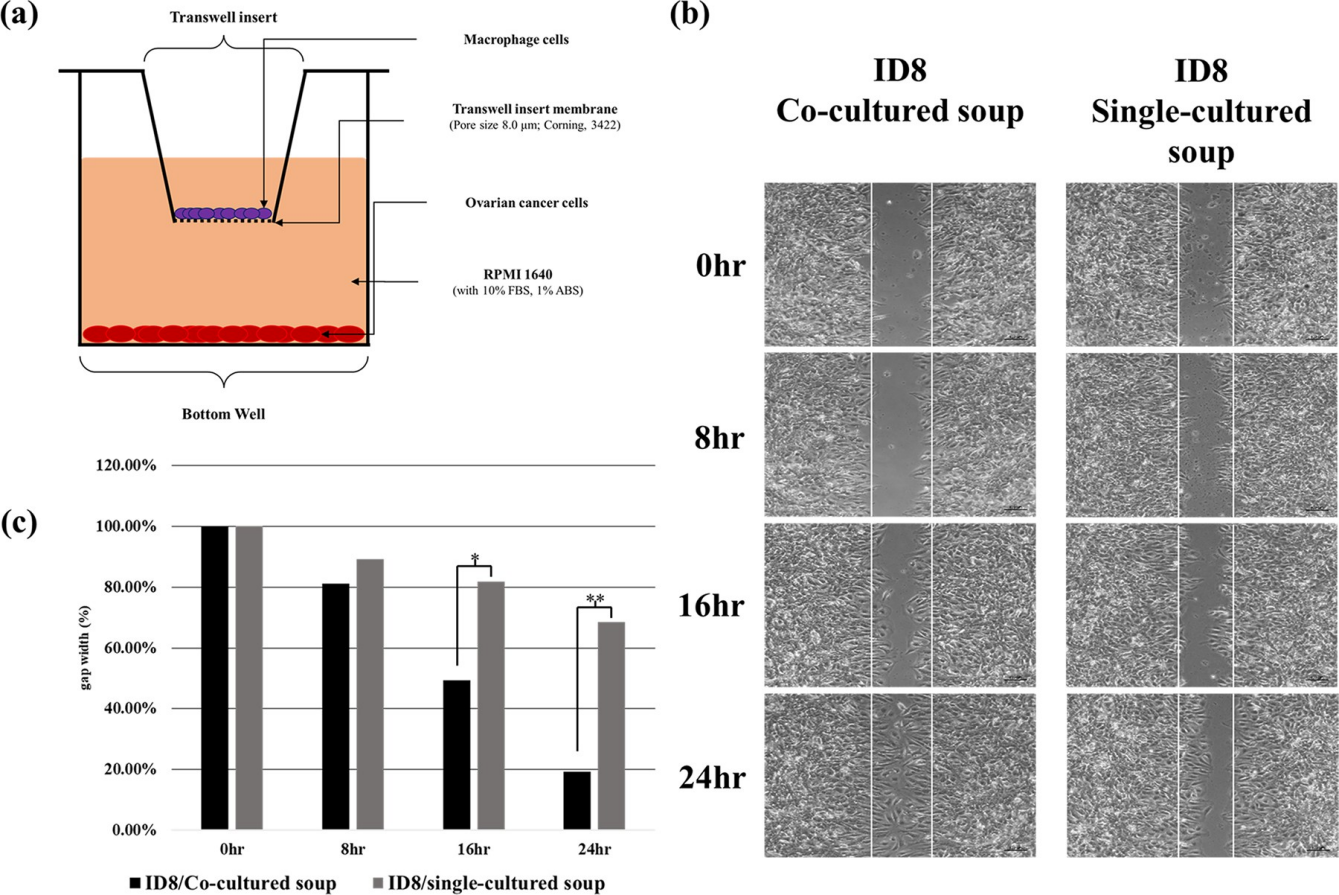
Schematic representation of the co-culture and wound healing assays with or without macrophages. (a) Depiction of the co-culture assay. (b) Representative images of the wound healing assay at 0, 8, 16, and 24 h time points. (c) Graphical representation of the wound healing assay results. Data are presented as the mean ± SEM of values from four independent experiments. * p < 0.001, ** p < 0.001.

We investigated the effects of macrophages on cell migration by performing an in vitro wound healing assay. Our results reveal that the co-cultured ID8 cells fill up the wound space faster than the single-cultured ID8 cells. This indicates that co-culture improves the migration and proliferation of ID8 cells. We further deduce that macrophages pose challenges in the elimination of cancer cells (Fig 1).

### Macrophages differentiate into pro-tumorigenic M2 phenotypes after co-culture with tumor cells

**Fig 2:**
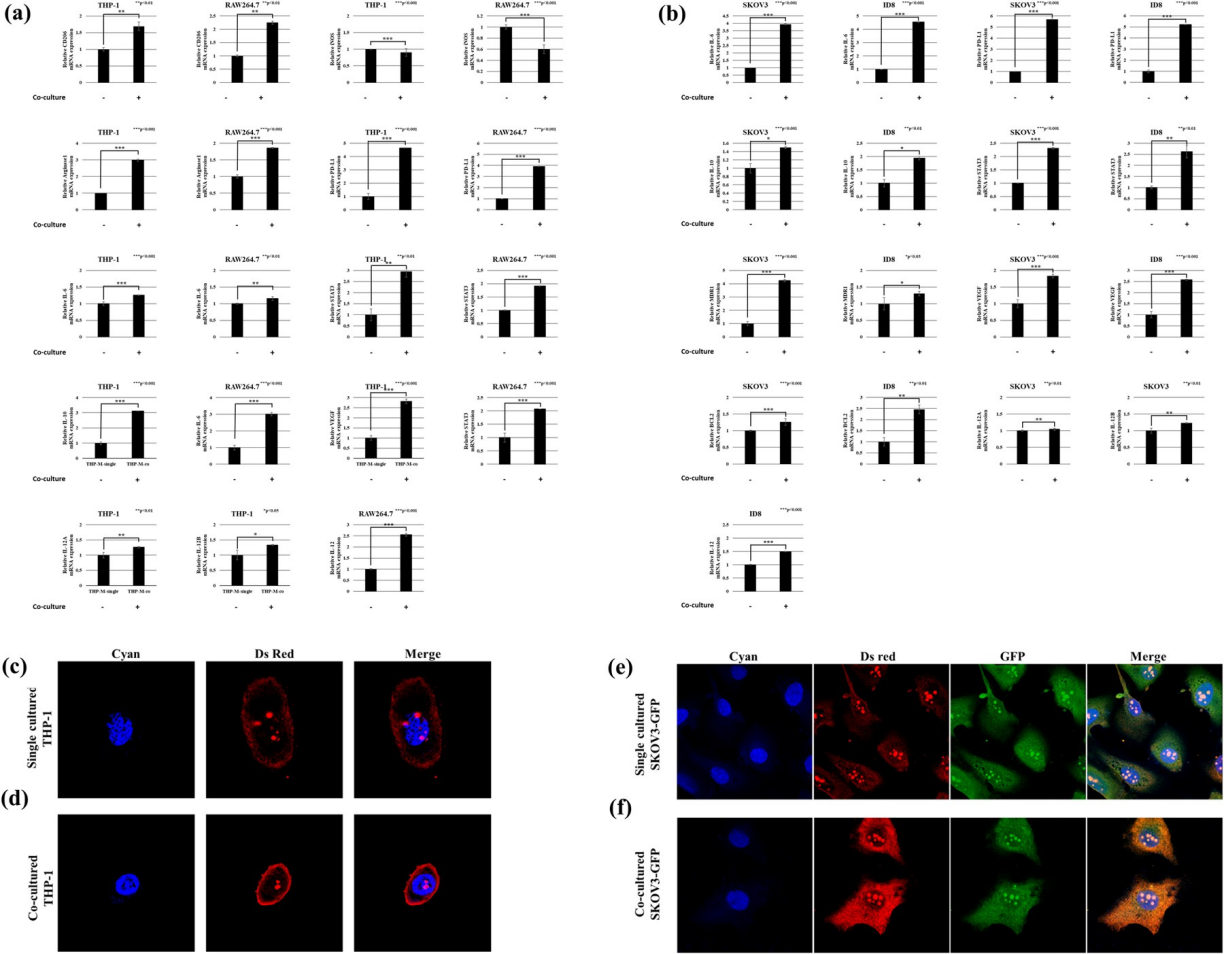
Gene expression in ovarian cancer cells and macrophages following co-culture. (a) mRNA expression of the M2/M1 markers in macrophages following co-culture. (b) mRNA expression of various genes related to chemoresistance and angiogenesis in co-cultured ovarian cancer cells. Immunofluorescence images of (c) single-cultured THP-1 cells, (d) co-cultured THP-1 cells, (e) single-cultured SKOV3-GFP cells, and (f) co-cultured SKOV3-GFP cells. The cyan color represents Hoechst 33342-stained nuclei observed at 440–480 nm with 358 nm excitation. The green color represents the green fluorescence-stained cytoplasm of SKOV3-GFP cells observed at 460–580 nm with 488 nm excitation. Ds Red color represents the fluorescence of PD-L1 observed at 500–700 nm with 480 nm excitation.

We analyzed the mRNA extracted from macrophages co-cultured with and without tumor cells via RT–PCR. The co-cultured macrophages exhibit upregulation of vascular endothelial cell growth factor (*VEGF*), signal transducer and activator of transcription 3 (*STAT3*), *PD-L1*, *CD206*, *IL-10*, and *ARG1*. Notably, these genes are immunosuppressive and pro-tumor markers in M2 macrophages. However, *IL-12* is upregulated in M1 phenotypes (Fig 2A).

Our data indicate that co-culturing promotes differential mRNA expression. For instance, the co-culture of THP-1 upregulates expression of *CD206*, *ARG1*, *IL-10*, *PD-L1*, *STAT3*, and *VEGF* (fold change > 1.5). However, *IL-6*, *IL-12A*, and *IL-12B* mRNA levels show only a marginal increase in expression (fold change < 1.5). In addition, we observe a small decrease in expression of *iNOS* mRNA levels (fold change < 1.5; Fig 2A). The RAW264.7 co-culture exhibits increased expression of *CD206*, *ARG1*, *IL-10*, *IL-12*, *PD-L1*, *STAT3*, and *VEGF* (fold change > 1.5). However, *IL-6* displays only a small observable increase in expression (fold change < 1.5), whereas *iNOS* shows a marginal reduction in expression (fold change < 1.5; Fig 2A). The mRNA expression of *IL-6*, *MDR1*, *PD-L1*, *STAT3*, and *VEFG* (fold change > 1.5) increases in the SKOV3 co-culture. However, *BCL2*, *IL-10*, *IL-12A*, and *IL-12B* exhibit only a small increase in expression levels (fold change < 1.5; Fig 2B). The ID8 co-culture exhibits an increase in *BCL2*, *IL-6*, *IL-10*, *PD-L1*, *STAT3*, and *VEFG* expression levels (fold change > 1.5). However, *MDR1* and *IL12* exhibit only a small increase in expression (fold change < 1.5; Fig 2B).

### Macrophages induce tumor cells to express immunosuppressive molecules

RT-PCR was used to analyze mRNA expression in tumor cells co-cultured with or without macrophages. The co-cultured ovarian cancer cells show increased expression of *PD-L1*, *VEGF*, *STAT3*, *IL-10*, *IL-6*, B-cell lymphoma 2 (*BCL2*), and *MDR1*. These genes are related to immunosuppression, chemoresistance, and angiogenesis (Fig 2B). Confocal microscopic images indicate that the expression of PD-L1 is markedly increased in both ovarian cancer cells and macrophages after co-culture (Fig 2C–2F).

### Macrophages induce chemoresistance against carboplatin in tumor cells

**Fig 3:**
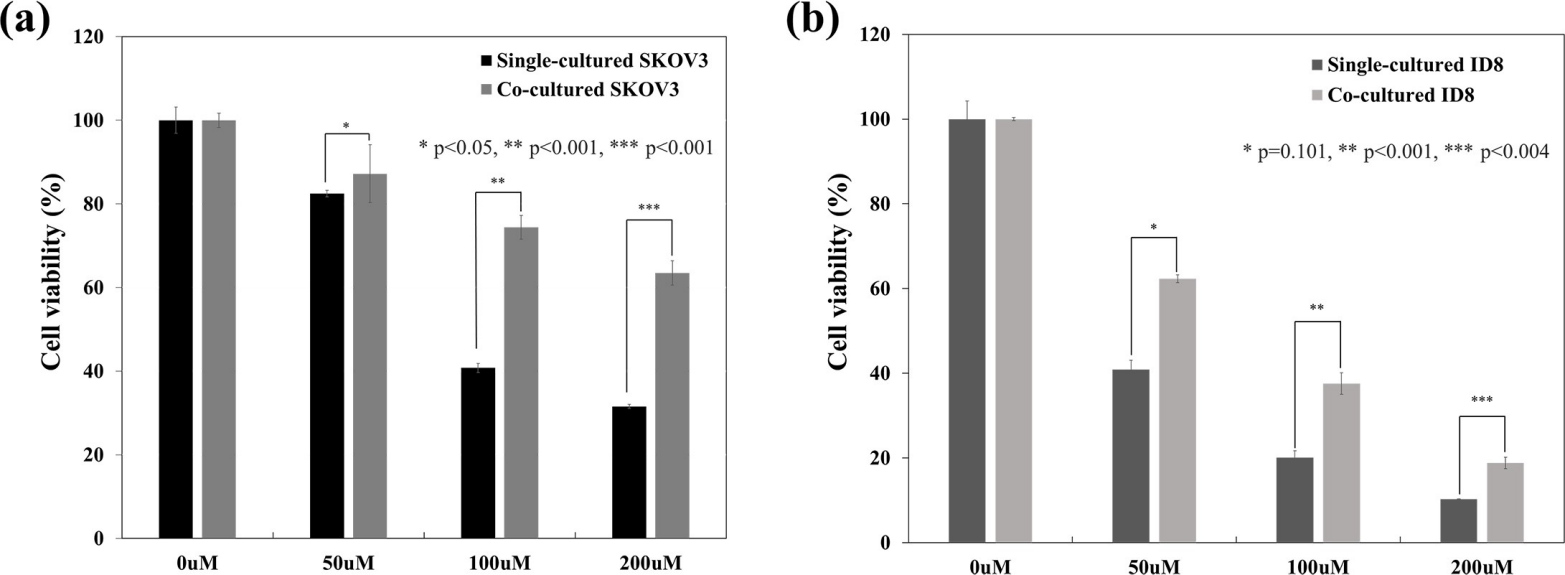
Viability of the single-cultured and co-cultured ovarian cancer cells following carboplatin treatment. (a) SKOV3 cells co-cultured with THP-1 cells. * p < 0.05, ** p < 0.001, *** p < 0.001. (b) ID8 cells co-cultured with RAW264.7 cells. * p = 0.101, ** p < 0.001, *** p < 0.004.

Cell viability results reveal that the chemosensitivity of ovarian cancer cells is reduced after co-culturing with macrophages. Moreover, we observe a significant difference in the chemosensitivity between 100 and 200 μM carboplatin concentrations (Fig 3).

### Silencing of PD-L1 in both macrophages and tumor cells reverses chemoresistance to carboplatin

**Fig 4:**
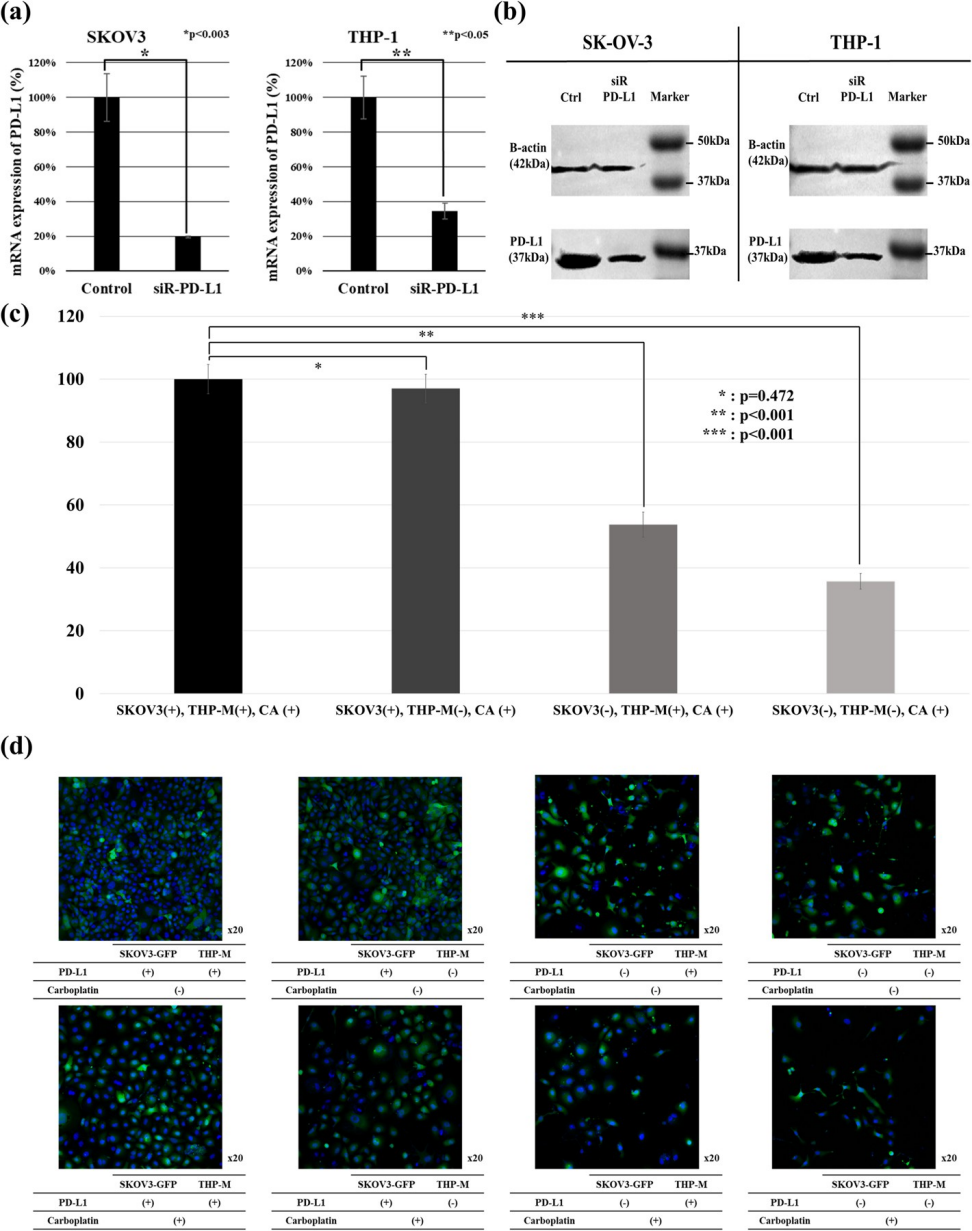
Silencing of *PD-L1* in human ovarian cancer and macrophage cell lines. (a) Silencing of *PD-L1* in SKOV3 and THP-1 cells. * p = 0.472, ** p < 0.001, *** p < 0.001. (b) Western blotting shows reduced PD-L1 protein expression. (c) Viability of *PD-L1*-silenced SKOV3 and THP-1 cells after carboplatin treatment. (d) Confocal fluorescence microscopic images of SKOV3 cells.

Treatment with *PD-L1* siRNA reduces the mRNA expression of *PD-L1* to 80.48% in SKOV3 cells and to 65.46% in THP-1 cells (Fig 4A). *PD-L1* siRNA also reduces the PD-L1 protein expression in SKOV3 cells and THP-1 cells (Fig 4B). Furthermore, *PD-L1* silencing increases the chemosensitivity of SKOV3 cells to carboplatin (p < 0.001). Increased chemosensitivity to carboplatin is the highest when *PD-L1* is silenced in both the SKOV3 and THP-1 cells (p < 0.001). *PD-L1* silencing in THP-1 cells alone is not statistically significant (p = 0.472) (Fig 4C). Next, we labeled ovarian cancer cells with GFP to evaluate their chemosensitivity. The intensity of GFP significantly decreases post-*PD-L1* silencing (Fig 4D).

### Association between PD-L1 expression and the OS and PFS of ovarian cancer patients

**Fig 5:**
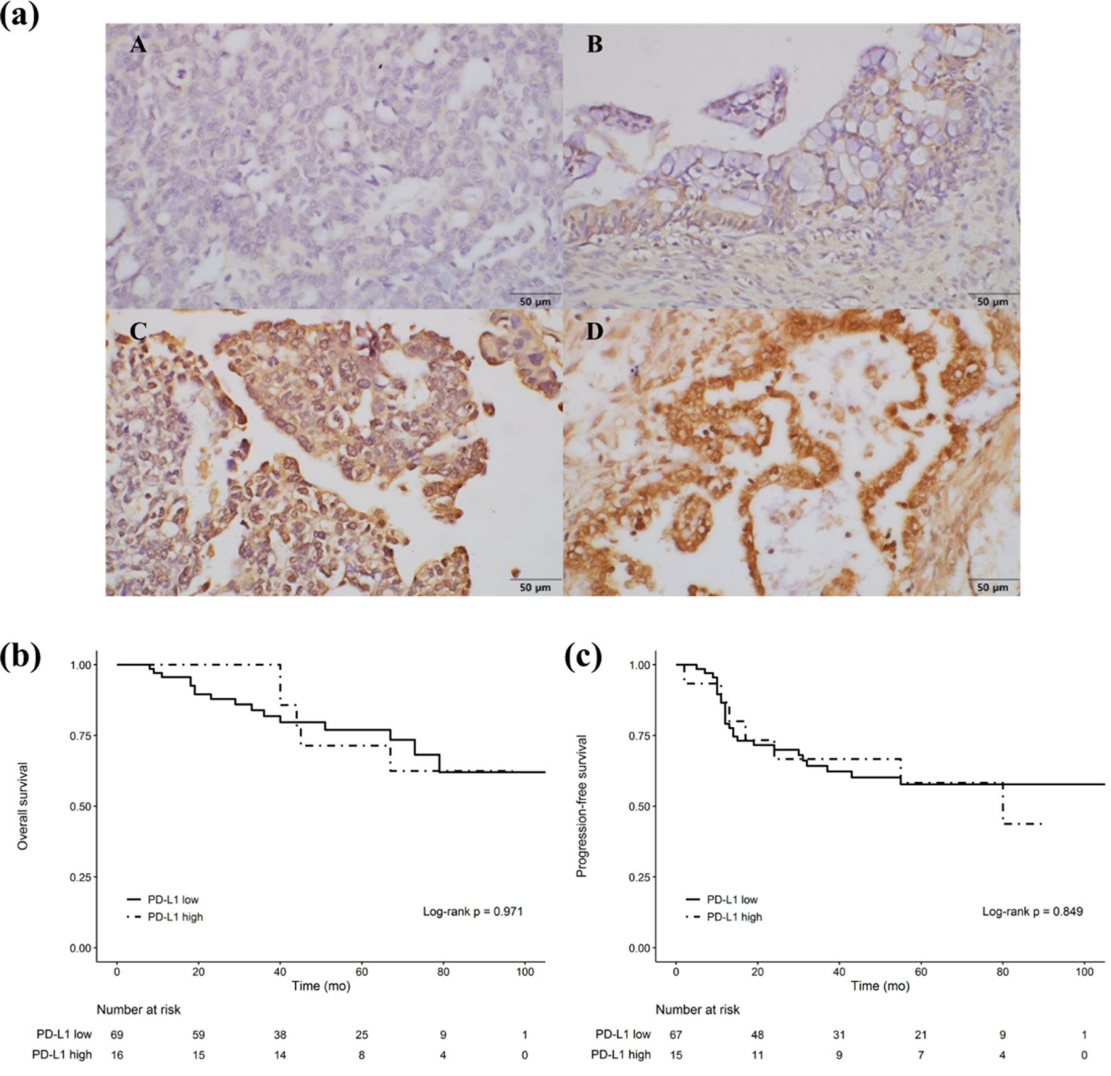
Immunohistochemical analysis of PD-L1 expression in ovarian cancer tissues and survival analysis of patients with low and high PD-L1 expression. (a) Representative immunohistochemical staining of PD-L1. (A) Negative staining, (B) weak staining, (C) moderate staining, and (D) strong staining. Analyses of (b) OS and (c) PFS based on PD-L1 expression.

We assessed PD-L1 expression in the tumor specimens of 85 ovarian cancer patients via IHC. Amongst these patients, 67 (79%) had PD-L1 low-positive tumors and 18 (21%) had PD-L1 high-positive tumors. No statistically significant association is observed between PD-L1 expression and the survival of both groups. Thus, we infer that PD-L1 expression is not a prognostic factor for ovarian cancer (Fig 5).

### Upregulation of CD68 in ovarian cancer cells and its association with OS and PFS

**Fig 6:**
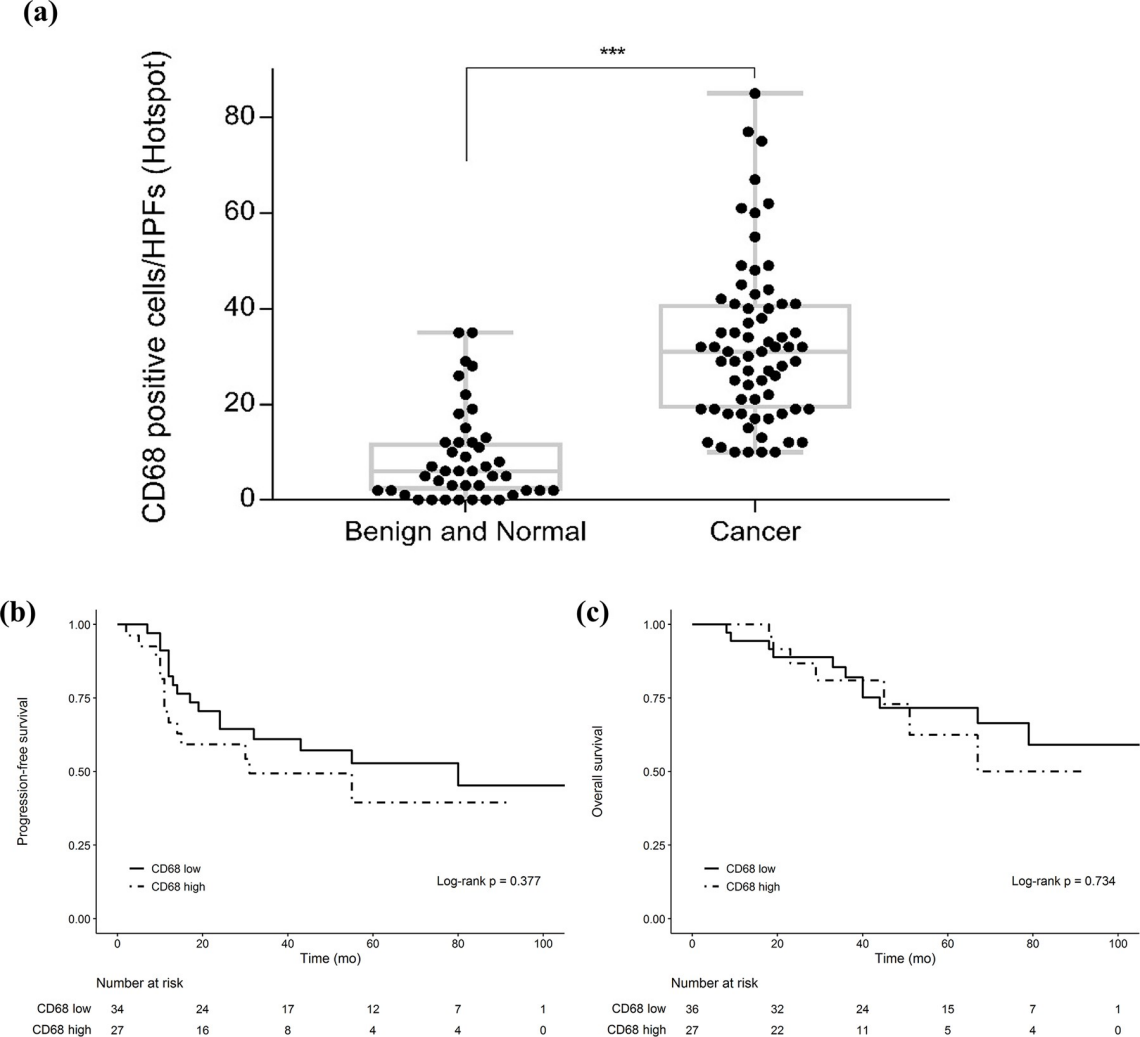
Difference in CD68 expression between normal or benign ovarian tissues and ovarian cancer tissues and survival analysis of patients with low and high CD68 expression. (a) CD68-positive cells in normal, benign, and cancer ovarian cells. * p < 0.05, ** p < 0.01, *** p < 0.001. Analysis of (b) OS and (c) PFS based on CD68 expression.

We compared the expression of CD68 in 85 cancer, 7 normal, and 35 benign ovarian tissues and found that the immunohistochemistry activity of CD68 is higher in the ovarian cancer tissues compared to that in the others. Tissues were divided into low and high groups based on the median value of CD68 expression in ovarian cancer tissues. The relationship between CD68 expression and survival outcomes was determined (Fig 6). No statistically significant association is observed between CD68 expression and the survival of both groups.

### Discussion

The primary reason for poor survival rates in ovarian cancer patients is the resistance to chemotherapeutic agents such as carboplatin and paclitaxel. Although the mechanism of chemoresistance in ovarian cancer is not completely understood, several studies have reported that factors such as the TME regulate this mechanism. In fact, the interplay between tumor cells and TAMs in the TME has been proposed as one of the reasons for therapeutic failure in cancer patients. The invasiveness of ovarian cancer cells is induced by various cytokines such as IL-10 and IL-6, which are secreted by the TAMs that have M2-like phenotypes and express *CD206* and arginase-1 *(ARG1)*. We determined that TAMs had increased expression of *IL-10*, *IL-6*, *CD206*, and *ARG1*, whereas ovarian cancer cells had increased expression of *IL-10*, *IL-6*, *VEGF*, *STAT3*, and *PD-L1*. Moreover, the co-cultured ovarian cancer cells exhibited faster migration and proliferation in the wound healing assay than the single-cultured ovarian cancer cells. The key pro-inflammatory factor IL-6 has been implicated to be involved in the tumor progression and MDR of various cancers [14–16]. It induces MDR by increasing the expression of MDR-1 and the apoptosis inhibitory protein Bcl-2. Furthermore, we previously found that certain cytokines and transcriptional factors, including IL-6, IL10, IL-27, STAT, and NF-kB, regulate PD-L1 expression in various cancers [17–21]. Macrophages activate STAT3 in tumor cells by expressing IL-6, thereby enhancing the proliferation and survival of malignant cells even during chemotherapeutic treatment [22]. We demonstrated that *IL-6*, *MDR-1*, and *BCL2* increased expression in the ovarian cancer cells co-cultured with macrophages.

The proteins PD-1 (encoded by *PDCD1*) and PD-L1 (encoded by *CD274*) are crucial factors for immune homeostasis [23]. However, the PD-1/PD-L1 axis in the TME is deregulated by cancer cells and TAMs to escape immune surveillance [24]. Therefore, PD-L1-expressing TAMs contribute to an immunosuppressive TME. We identified the robust expression of PD-L1 in both the tumor cells and TAMs following co-culture. Remarkably, our Transwell co-culture assay revealed that ovarian cancer cells became resistant to carboplatin after co-cultivation with macrophages. Although the role of the PD-L1 and PD-1 interaction in regulating T-cell suppression has been well established, little is known about PD-L1 signaling in ovarian cancer chemotherapy resistance. Thus, we hypothesized that PD-L1 in both ovarian cancer cells and TAMs might be associated with carboplatin resistance. In our experiments, *PD-L1* silencing in SKOV3 cells increased their chemosensitivity to carboplatin. Moreover, when *PD-L1* was silenced in both SKOV3 and THP-1 cells, the chemosensitivity to carboplatin was the highest. Therefore, we concluded that PD-L1 knockdown in both ovarian cancer cells and macrophages increased the sensitivity of the ovarian cancer cells to carboplatin. Although blocking the PD-1 and PD-L1 axis is an attractive target for cancer treatment, many preclinical and clinical studies have concentrated on the relationship between PD-L1 and immunosuppression, but not chemoresistance. It has been reported that microRNA-197 enhances PD-L1 expression and chemoresistance in lung cancer cells [25]. Xu et al. (2016) indicated that microRNA-424 treatment reduces PD-L1 expression by directly binding to the 3′-untranslated region of human and mouse genes and reverses chemoresistance via the T-cell immune response [26]. Furthermore, Zhang, et al. [13] reported that alterations in PD-L1 levels can regulate chemoresistance in lung cancer. They also reported that upregulation of PD-L1 possibly occurs via the activated PI3K/AKT signaling pathway [27].

Anti-PD-1 or PD-L1 inhibition induces the polarization of macrophages to the M1 phenotype, subsequently rescuing their phagocytic function and enhancing their antitumor effects [28–31]. In our study, the knockdown of PD-L1 in macrophages reduced the expression of *iNOS*, *VEGF*, *IL-6*, and *IL-10*.

While PD-L1 expression is associated with poor patient prognosis for a variety of solid tumors, including cervical cancer, its prognostic value in ovarian cancer remains controversial. Wang [32] performed a meta-analysis of 12 studies, involving 1630 ovarian cancer patients, by conducting an IHC analysis of PD-L1 expression to investigate the prognostic impact of PD-L1 in ovarian cancer. Wang suggested that PD-L1 expression is not linked to the tumor grade, clinical stage, lymph node status, tumor histology, and patient OS and PFS [32]. Our results are consistent with these observations, as we did not observe any association between PD-L1 expression and PFS or OS. Our study indicates that interactions between ovarian cancer cells and macrophages confer ovarian cancer cells with migratory, proliferative, and chemotherapy-resistant properties. In contrast, macrophages polarize to the M2 phenotype following co-culture. Nonetheless, both tumor cells and macrophages show robust PD-L1 expression upon co-culture. However, PD-L1 knockdown reverses the chemoresistance of ovarian cancer cells to carboplatin.

This study has the limitation of a small sample size. Additionally, the expression of PD-L1 in ovarian cancer tissues was not found to have any association with patient survival. However, our study offers a possible mechanism for PD-L1-related chemoresistance in ovarian cancer cells and macrophages. Further research is required to completely elucidate this mechanism for the discovery of novel therapeutic strategies.

## Supporting information

S1 File (XLSX)

## References

[1] Sung H, Ferlay J, Siegel RL, Laversanne M, Soerjomataram I, Jemal A, et al. Global cancer statistics 2020: GLOBOCAN estimates of incidence and mortality worldwide for 36 cancers in 185 countries. CA Cancer J Clin. 2021;71(3): 209–249. doi: 10.3322/caac.21660 33538338

[2] Obeid E, Nanda R, Fu Y-X, Olufunmilayo OI. The role of tumor-associated macrophages in breast cancer progression (review). Int J Oncol. 2013;43(1): 5–12. doi: 10.3892/ijo.2013.1938 23673510 PMC3742164

[3] Huang Y, Ge W, Zhou J, Gao B, Qian X, Wang W. The role of tumor associated macrophages in hepatocellular carcinoma. J Cancer. 2021;12(5): 1284–1294. doi: 10.7150/jca.51346 33531974 PMC7847664

[4] Schweer D, McAtee A, Neupane K, Richards C, Ueland F, Kolesar J. Tumor-associated macrophages and ovarian cancer: implications for therapy. Cancers. 2022;14(9): 2220. doi: 10.3390/cancers14092220 35565348 PMC9101750

[5] Rihawi K, Ricci AD, Rizzo A, Brocchi S, Marasco G, Pastore LV, et al. Tumor-associated macrophages and inflammatory microenvironment in gastric cancer: novel translational implications. Int J Mol Sci. 2021;22(8): 3805. doi: 10.3390/ijms22083805 33916915 PMC8067563

[6] Gordon S, Martinez FO. Alternative activation of macrophages: mechanism and functions. Immunity. 2010;32: 593–604. doi: 10.1016/j.immuni.2010.05.007 20510870

[7] Hagemann T, Wilson J, Burke F, Kulbe H, Li NF, Plüddemann A, et al. Ovarian cancer cells polarize macrophages toward a tumor-associated phenotype. J Immunol. 2006;176(8): 5023–5032. doi: 10.4049/jimmunol.176.8.5023 16585599

[8] Duluc D, Delneste Y, Tan F, Moles MP, Grimaud L, Lenoir J, et al. Tumor-associated leukemia inhibitory factor and IL-6 skew monocyte differentiation into tumor-associated macrophage-like cells. Blood. 2007;110(13): 4319–4330. doi: 10.1182/blood-2007-02-072587 17848619

[9] Nowak M, Klink M. The role of tumor-associated macrophages in the progression and chemoresistance of ovarian cancer. Cells. 2020;9(5): 1299. doi: 10.3390/cells9051299 32456078 PMC7290435

[10] Yuan X, Zhang J, Li D, Mao Y, Mo F, Du W, et al. Prognostic significance of tumor-associated macrophages in ovarian cancer: a meta-analysis. Gynecol Oncol. 2017;147(1): 181–187. doi: 10.1016/j.ygyno.2017.07.007 28698008

[11] Liu R, Hu R, Zeng Y, Zhang W, Zhou H-H. Tumour immune cell infiltration and survival after platinum-based chemotherapy in high-grade serous ovarian cancer subtypes: a gene expression-based computational study. EBioMedicine. 2020;51: 102602. doi: 10.1016/j.ebiom.2019.102602 31911269 PMC6948169

[12] Dijkgraaf EM, Heusinkveld M, Tummers B, Vogelpoel LTC, Goedemans R, Jha V, et al. Chemotherapy alters monocyte differentiation to favor generation of cancer-supporting M2 macrophages in the tumor microenvironment. Cancer Res. 2013;73(8): 2480–2492. doi: 10.1158/0008-5472.CAN-12-3542 23436796

[13] Zhang P, Ma Y, Lv C, Huang M, Li M, Dong B, et al. Upregulation of programmed cell death ligand 1 promotes resistance response in non-small-cell lung cancer patients treated with neo-adjuvant chemotherapy. Cancer Sci. 2016;107(11): 1563–1571. doi: 10.1111/cas.13072 27581532 PMC5132280

[14] Iliopoulos D, Hirsch HA, Wang G, Struhl K. Inducible formation of breast cancer stem cells and their dynamic equilibrium with non-stem cancer cells via IL6 secretion. Proc Natl Acad Sci U S A. 2011;108(4): 1397–1402. doi: 10.1073/pnas.1018898108 21220315 PMC3029760

[15] Rokavec M, Öner MG, Li H, Jackstadt R, Jiang L, Lodygin D, et al. IL-6R/STAT3/miR-34a feedback loop promotes EMT-mediated colorectal cancer invasion and metastasis. J Clin Invest. 2014;124(4): 1853–1867. doi: 10.1172/JCI73531 24642471 PMC3973098

[16] Ghandadi M, Sahebkar A. Interleukin-6: a critical cytokine in cancer multidrug resistance. Curr Pharm Des. 2016;22(5): 518–526. doi: 10.2174/1381612822666151124234417 26601970

[17] Xu L, Chen X, Shen M, Yang DR, Fang L, Weng G, et al. Inhibition of IL-6-JAK/Stat3 signaling in castration-resistant prostate cancer cells enhances the NK cell-mediated cytotoxicity via alteration of PD-L1/NKG2D ligand levels. Mol Oncol. 2018;12(3): 269–286. doi: 10.1002/1878-0261.12135 28865178 PMC5830627

[18] Jiang C, Yuan F, Wang J, Wu L. Oral squamous cell carcinoma suppressed antitumor immunity through induction of PD-L1 expression on tumor-associated macrophages. Immunobiology. 2017;222(4): 651–657. doi: 10.1016/j.imbio.2016.12.002 28017495

[19] Carbotti G, Barisione G, Airoldi I, Mezzanzanica D, Bagnoli M, Ferrero S, et al. IL-27 induces the expression of IDO and PD-L1 in human cancer cells. Oncotarget. 2015;6(41): 43267–43280. doi: 10.18632/oncotarget.6530 26657115 PMC4791231

[20] Bi XW, Wang H, Zhang WW, Wang JH, Liu WJ, Xia ZJ, et al. PD-L1 is upregulated by EBV-driven LMP1 through NF-κB pathway and correlates with poor prognosis in natural killer/T-cell lymphoma. J Hematol Oncol. 2016;9(1): 109. 27737703 10.1186/s13045-016-0341-7 PMC5064887

[21] Jin X, Ding D, Yan Y, Li H, Wang B, Ma L, et al. Phosphorylated RB promotes cancer immunity by inhibiting NF-κB activation and PD-L1 expression. Mol Cell. 2019;73(1): 22-35.e6. 10.1016/j.molcel.2018.10.034 PMC8968458 30527665

[22] Ruffell B, Coussens LM. Macrophages and therapeutic resistance in cancer. Cancer Cell. 2015;27(4): 462–472. doi: 10.1016/j.ccell.2015.02.015 25858805 PMC4400235

[23] Akinleye A, Rasool Z. Immune checkpoint inhibitors of PD-L1 as cancer therapeutics. J Hematol Oncol. 2019;12: 92. doi: 10.1186/s13045-019-0779-5 31488176 PMC6729004

[24] Li X, Shao C, Shi Y, Han W. Lessons learned from the blockade of immune checkpoints in cancer immunotherapy. J Hematol Oncol. 2018;11(1): 31. doi: 10.1186/s13045-018-0578-4 29482595 PMC6389077

[25] Fujita Y, Yagishita S, Hagiwara K, Yoshioka Y, Kosaka N, Takeshita F, et al. The clinical relevance of the miR-197/CKS1B/STAT3-mediated PD-L1 network in chemoresistant non-small-cell lung cancer. Mol Ther. 2015;23(4): 717–727. doi: 10.1038/mt.2015.10 25597412 PMC4395779

[26] Xu S, Tao Z, Hai B, Liang H, Shi Y, Wang T, et al. miR-424(322) reverses chemoresistance via T-cell immune response activation by blocking the PD-L1 immune checkpoint. Nat Commun. 2016;7: 11406. doi: 10.1038/ncomms11406 27147225 PMC4858750

[27] Zhang X, Zeng Y, Qu Q, Zhu J, Liu Z, Ning W, et al. PD-L1 induced by IFN-γ from tumor-associated macrophages via the JAK/STAT3 and PI3K/AKT signaling pathways promoted progression of lung cancer. Int J Clin Oncol. 2017;22: 1026–1033. 28748356 10.1007/s10147-017-1161-7

[28] Chen D., Xie J, Fiskesund R, Dong W, Liang X, Lv J, et al. Chloroquine modulates antitumor immune response by resetting tumor-associated macrophages toward M1 phenotype. Nat Commun. 2018;9(1): 873. doi: 10.1038/s41467-018-03225-9 29491374 PMC5830447

[29] Meng Y, Qu Y, Wu W, Chen L, Sun L, Tai G, et al. Galactan isolated from Cantharellus cibarius modulates antitumor immune response by converting tumor-associated macrophages toward M1- like phenotype. Carbohydr Polym. 2019; 226: 115295. doi: 10.1016/j.carbpol.2019.115295 31582086

[30] Ma Q, Gu JT, Wang B, Feng J, Yang L, Kang XW, et al. PlGF signaling and macrophage repolarization contribute to the anti-neoplastic effect of metformin. Eur J Pharmacol. 2019;863: 172696. doi: 10.1016/j.ejphar.2019.172696 31562866

[31] Rodell CB, Arlauckas SP, Cuccarese MF, Garris CS, Li R, Ahmed MS, et al. TLR7/8-agonist-loaded nanoparticles promote the polarization of tumour-associated macrophages to enhance cancer immunotherapy. Nat Biomed Eng. 2018;2(8): 578–588. doi: 10.1038/s41551-018-0236-8 31015631 PMC6192054

[32] Wang L. Prognostic effect of programmed death-ligand 1 (PD-L1) in ovarian cancer: a systematic review, meta-analysis and bioinformatics study. J Ovarian Res. 2019;12(1): 37. doi: 10.1186/s13048-019-0512-6 31039792 PMC6492430

